# Protection of Human Lens Epithelial Cells from Oxidative Stress Damage and Cell Apoptosis by KGF-2 through the Akt/Nrf2/HO-1 Pathway

**DOI:** 10.1155/2022/6933812

**Published:** 2022-02-17

**Authors:** Shuyu Liu, Zi Jin, Ruyue Xia, Zhuoni Zheng, Yi Zha, Qiang Wang, Xinbei Wan, Hui Yang, Jianqiu Cai

**Affiliations:** ^1^Department of Ophthalmology, The Second Affiliated Hospital and Yuying Children's Hospital of Wenzhou Medical University, Wenzhou 325000, China; ^2^School of Pharmaceutical Science, Wenzhou Medical University, Wenzhou 325000, China; ^3^Department of Ophthalmology, Ruian People's Hospital, Wenzhou 325000, China; ^4^Department of Epidemiology, Biostatistics and Occupational Health, McGill University, Montreal, Quebec, Canada H3A 1G1

## Abstract

Oxidative stress exerts a significant influence on the pathogenesis of various cataracts by inducing degradation and aggregation of lens proteins and apoptosis of lens epithelial cells. Keratinocyte growth factor−2 (KGF-2) exerts a favorable cytoprotective effect against oxidative stress *in vivo* and *in vitro*. In this work, we investigated the molecular mechanisms of KGF-2 against hydrogen peroxide- (H_2_O_2_-) induced oxidative stress and apoptosis in human lens epithelial cells (HLECs) and rat lenses. KGF-2 pretreatment could reduce H_2_O_2_-induced cytotoxicity as well as reactive oxygen species (ROS) accumulation. KGF-2 also increases B-cell lymphoma-2 (Bcl-2), quinine oxidoreductase-1 (NQO-1), superoxide dismutase (SOD2), and catalase (CAT) levels while decreasing the expression level of Bcl2-associated X (Bax) and cleaved caspase-3 in H_2_O_2_-stimulated HLECs. LY294002, the phosphatidylinositol-3-kinase (PI3K)/Akt inhibitor, abolished KGF-2's effect to some extent, demonstrating that KGF-2 protected HLECs via the PI3K/Akt pathway. On the other hand, KGF-2 activated the Nrf2/HO-1 pathway by regulating the PI3K/Akt pathway. Silencing nuclear factor erythroid 2-related factor 2 (Nrf2) by targeted-siRNA and inhibiting heme oxygenase-1 (HO-1) through zinc protoporphyrin IX (ZnPP) significantly decreased cytoprotection of KGF-2. Furthermore, as revealed by lens organ culture assays, KGF-2 treatment decreased H_2_O_2_-induced lens opacity in a concentration-dependent manner. As demonstrated by these data, KGF-2 resisted H_2_O_2_-mediated apoptosis and oxidative stress in HLECs through Nrf2/HO-1 and PI3K/Akt pathways, suggesting a potential protective effect against the formation of cataracts.

## 1. Introduction

Cataract, primarily age-related, is the main reason for visual impairment and blindness across the world [1]. Currently, cataractous lens removal surgery is the only effective remedy. However, it still presents the risk of surgical complications such as posterior capsular opacification, which gives rise to the second loss of clear vision [2]. Furthermore, no efficient clinical therapies have been proposed to prevent the formation of cataracts until now. Hence, pharmacological intervention to maintain the transparency of the lens is vital.

Oxidative stress, mediated by reactive oxygen species (ROS), where DNA, proteins, lipids, and cells are damaged, is the leading contributor to the formation of cataracts [3, 4]. In addition, oxidative stress triggers apoptosis for human lens epithelial cells (HLECs) by altering the internal environment. It is considered the common molecular basis for cataract initiation as well as progression [5]. Hydrogen peroxide (H_2_O_2_) is a nonradical member in ROS, which damages ion pump activity in HLECs [6, 7]. Raising H_2_O_2_ levels of aqueous humor are found among patients suffering cataracts which cause lens opacification *in vitro* [8]. H_2_O_2_ has gained extensive uses in inducing oxidative stress *in vitro* [9–11]. Subsequently, protecting HLECs against H_2_O_2_-induced oxidative stress and apoptosis is a potential solution to postpone cataract development.

Since no effective therapeutic drugs have been proposed for halting the formation of the cataractous lens, developing a pharmacological therapy to postpone cataract progression and ameliorate lens transparency is imperative. Evidence suggests that growth factors help prevent injury from various causes, including H_2_O_2_, radiation, and bleomycin [12–14]. In addition, recent studies demonstrate that growth factors possibly reduce oxidant-induced lung damage by suppressing apoptosis [15]. However, few studies have confirmed the function of growth factors in HLECs.

Keratinocyte growth factor−2 (KGF-2), also known as fibroblast growth factor−10 (FGF-10), is a critical member of the FGF family. As a multifunctional growth factor, KGF-2 plays an important role in developing various organs and tissues, including the eye [16]. For example, KGF-2 null murine embryos demonstrate agenesis in ocular glands-extra orbital and intraorbital lacrimal glands and Harderian gland [17]. Several studies have confirmed that KGF-2 can decrease oxidant-induced cell injury, inhibit cell apoptosis, and regulate cell homeostasis in numerous organs including the kidney, lung, and spinal cord [18–21]. The KGF-2 intraocular medication has been proved to be safe. Previous studies have shown that rhKGF-2 eye drop application in the long run in the rabbit corneas does neither result in any apparent systemic effects nor cause toxicity to the intraocular tissues [22]. Furthermore, the regulatory effect of KGF-2 on the PI3K/Akt pathway is confirmed [23, 24]. As a survival pathway, the PI3K/Akt pathway modulates cell proliferation, migration, metabolism, and differentiation within diverse disorders [25]. According to increasing evidence, Akt activation can protect HLECs from oxidative stress damage and apoptosis [26, 27].

Nrf2 is a transcription factor that can regulate the expression of antioxidants, transporters, autophagy-related proteins, and enzymes involved in metabolism and detoxification (like heme oxygenase-1 (HO-1)) [28]. Studies have also suggested that PI3K/Akt signaling can regulate Nrf2 expression [29]. Previous studies have elucidated that Nrf2 exerts a dominant influence on protecting the lens from oxidative stress [30]. The activation of Nrf2 may lower oxidative stress and prevent cataract formation [31]. As a result, Nrf2 may play the role of a promising therapeutic target for cataract treatment.

Therefore, we adopted H_2_O_2_-mediated HLECs and rat lenses as study models and for the first time demonstrated that KGF-2 may protect HLECs against oxidative stress-mediated apoptosis via the Akt/Nrf2/HO-1 pathway and may prove beneficial for treating cataracts associated with oxidative stress.

## 2. Materials and Methods

### 2.1. Reagents and Antibodies

KGF-2 was provided by the School of Pharmacy, Wenzhou Medical University (Wenzhou, China). Antibodies of p-Akt (#4060), Akt (#4685), cleaved caspase-3 (#9664), Bcl-2 (#4223), and Bax (#2772) were provided by Cell Signaling Technology (Danvers, MA, USA). Antibodies of Nrf2 (ab137550) and the PI3K/Akt inhibitor LY294002 (ab120243) were provided by Abcam (Cambridge, MA, USA). Antibodies of *β*-actin (20536-1-AP), HO-1 (27282-1-AP), NQO-1 (11451-1-AP), CAT (21260-1-AP), SOD2 (24127-1-AP), and horseradish peroxidase-conjugated goat antimouse/rabbit secondary antibodies (SA00001-1 or SA00001-2) came from ProteinTech (Rosemont, USA). Hydrogen peroxide (H_2_O_2_, #349887), zinc protoporphyrin IX (ZnPP, #282820) and 3-(4,5-dimethylthiazol-2-yl)-2,5-diphenyltetrazolium bromide (MTT, M2128), and dimethyl sulfoxide (DMSO, D8418) came from Sigma-Aldrich (St. Louis, MO, USA). The reagents of cell culture were obtained from Gibco (Grand Island, NY, USA). The fluorescent dyes 2′,7′-dichlorodihydrofluorescein diacetate (DCFH-DA, S0033) and Annexin V-FITC/PI apoptosis detection kit (C1062L) were provided by Beyotime (Shanghai, China). Nrf2-small interfering RNA and fluorescein-conjugated control siRNA (sc-37030 and sc-36869) were provided by Santa Cruz Biotechnology Inc. (CA, USA).

### 2.2. Cell Culture and Treatment

HLECs obtained from American Type Culture Collection (ATCC, Manassas, VA, USA) were incubated by Modified Dulbecco's Eagle's medium (DMEM) supplemented with heat-inactivated (56°C, 0.5 h) 15% fetal bovine serum (FBS), 100 U/mL penicillin, plus 100 mg/mL streptomycin in humidified 5% CO_2_ at 37°C. Cells underwent subculture regularly every 2 or 3 days. When reaching 70% confluence, cells received treatment immediately using the indicated H_2_O_2_ concentration for 12 h or pretreatment at various KGF-2 concentrations for 2 h before H_2_O_2_ treatment. For continually determining how PI3K/Akt activation affected oxidative injury, cells first received pretreatment with PI3K inhibitor LY294002 (20 *μ*M) for 2 h.

### 2.3. Cell Viability Measurement

The MTT assay was performed to assess cell viability. HLECs (2 × 10^4^/well) were inoculated onto 96-well plates. Cells were incubated at varying H_2_O_2_ concentrations (0, 12.5, 25, 50, 100, 200, 400, and 800 *μ*M) for 6, 9, 12, 18 and 24 h or pretreated by KGF-2 for 24 h at varying concentrations (0, 12.5, 25, 50, 100, and 200 *μ*g/mL) and sometimes pretreated by KGF-2 for 2 h at varying concentrations (0, 12.5, 25, 50, 100, and 200 *μ*g/mL); then, H_2_O_2_ (100 *μ*M) was added to treat cells for 12 h. At times, cells underwent pretreatment initially with 10 *μ*M zinc protoporphyrin IX (ZnPP, the HO-1 inhibitor) for 30 min, followed by 2 h treatment using 50 *μ*g/mL KGF-2 prior to 12 h incubation using H_2_O_2_ (100 *μ*M). HLECs underwent 4 h incubation using MTT solution (20 *μ*L, 5 mg/mL) under 37°C. Besides, the culture medium was substituted by 150 *μ*L Thereafter, DMSO was added for dissolving formazan crystals following incubation. Afterwards, optical density was calculated with 490 nm wavelength with a microplate reader (Molecular Devices, Sunnyvale, CA, USA).

### 2.4. Apoptosis Analysis

Flow cytometric analysis was used to measure cell apoptosis in accordance with the protocol offered by Annexin V-FITC/PI apoptosis kit. Briefly, we exposed HLECs to H_2_O_2_ (100 *μ*M) for 12 h or pretreated with 12.5 or 50 *μ*g/mL KGF-2 for 2 h and then treated with H_2_O_2_ (100 *μ*M) for another 12 h. In total, Annexin V-FITC (5 *μ*L) together with PI (10 *μ*L), PI was added, followed by 15 min tube incubation in dark. The apoptotic rate was later quantitatively analyzed with the use of a flow cytometer (FACScan, Becton-Dickinson, USA).

### 2.5. Determination of Intracellular Redox State

Intracellular ROS levels were detected with flow cytometry. We inoculated HLECs (4 × 10^5^/well) into the 6-well plate. On the next day, we exposed cells to H_2_O_2_ (100 *μ*M) for 12 h or pretreated with 12.5 or 50 *μ*g/mL KGF-2 for 2 h and then treated them with H_2_O_2_ (100 *μ*M) for another 12 h. Afterwards, a DCFH-DA reagent (10 *μ*M/L) was utilized to incubate cells under 37°C for 0.5 h. ROS generation was finally evaluated using fluorescence microscopy and a FACScan flow cytometer (FACScan, Becton-Dickinson, USA).

### 2.6. Western Blot Analysis

HLECs treated above were collected and lysed in RIPA buffer via phosphatase (Applygen, P1260) and protease (Boster, AR0101/AR0103) inhibitors. The lysate received centrifugation at 12000 g for 20 min, and cytosol proteins were extracted. Bicinchoninic acid (BCA) reagent determined the protein concentration. Proteins were isolated using 12–15% SDS-PAGE before transfer on PVDF membranes. Thereafter, we blocked membranes using 5% skimmed milk and cultured them using primary antibodies, including *p*-Akt (1 : 1000), Akt (1 : 1000), Nrf2 (1 : 1000), NQO1 (1 : 1000), HO-1 (1 : 1000), SOD2 (1 : 2000), CAT (1 : 1000), Bcl-2 (1 : 1000), Bax (1 : 1000), and *β*-actin (1 : 10000), under 4°C overnight, followed by another 1 h horseradish peroxidase-conjugated-labeled secondary antibody incubation on the following day. Visualization for signals and band intensity was done with chemiluminescence in the gel imaging system (Bio-Rad Laboratories, Hercules, CA, USA).

### 2.7. Nrf2 siRNA Transfection

The siRNA targeting the Nrf2(sc-37030) was adopted for knocking down Nrf2 levels. We analyzed transfection efficiency using fluorescein-labeled control siRNA(sc-36869). HLECs were first seeded into a 6-well plate, followed by incubation under 5% CO_2_ and 37°C conditions till then reached 70–80% confluence. Then, in terms of each transfection, we diluted Nrf2 or control siRNA (2 *μ*L) and transfection reagent (2 *μ*L, sc-29528) in the siRNA transfection medium (100 *μ*L, sc-36868; Santa Cruz, CA, USA), separately. Then, we combined dilutions to incubate under ambient temperature for 0.5 h. After that, cells were transfected for 12 h and subsequently treated with 50 *μ*g/mL KGF-2 for 2 h before the treatment with 100 *μ*M H_2_O_2_ for 12 h.

### 2.8. Lens Organ Culture and Treatment

Male SD rats were obtained from the Charles River (Beijing, China). The Institutional Animal Experimentation Ethics Committee of the Wenzhou Medical University approved all animal protocols in the current work. Rat eyes were firstly extracted and stored within the heated (37°C) mammalian normal saline. We then cultured fresh transparent lenses within the medium that contained 0.1% BSA and gentamicin (50 mg/mL) under 5% CO_2_ and 37°C conditions. Lenses were treated with KGF-2 at the concentration of 50 and 100 *μ*g/mL for 2 h at 37°C. Then, with the addition of 100 *μ*M H_2_O_2_ for 12 h, lenses were detected via the stereomicroscope and shot in a black gridline background, thus recording the opacity development course. Further, lenses per group were rinsed in cold saline, placed on a filter paper for drying, dissected using Vannas scissors, and finally milled above the ice. Eventually, the tissue was spun down inside the refrigerated centrifuge at 1000 rpm for 10 min. Supernatants underwent lysis treatment above the ice for 20 min through radioimmunoprecipitation assay (RIPA) buffer with protease inhibitor cocktail and centrifugation at 12000 g for 20 min. Variations of indicated protein expression levels were assessed through western blotting as described above.

### 2.9. Statistical Analysis

Data used in the current experiment were indicated by mean ± SD. GraphPad Prism 6 (GraphPad Software, San Diego, CA, USA) was employed for statistical analyses. ANOVA was conducted to analyze significance. *p* values below 0.05 suggested statistical significance.

## 3. Results

### 3.1. Effects of H_2_O_2_ and KGF-2 on HLEC's Viability

**Figure 1:**
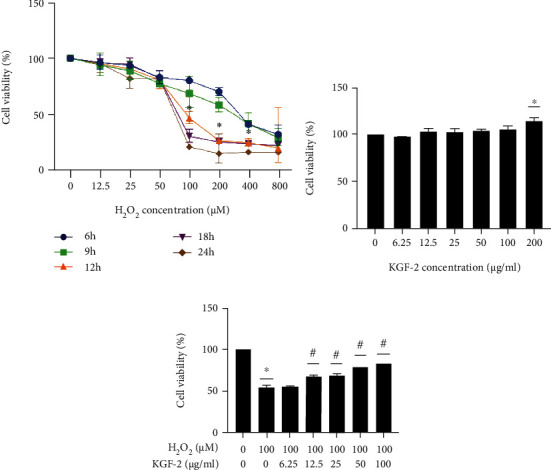
KGF-2 relieved H_2_O_2_-induced viability decrease in HLECs. (a) Exposure of HLECs to various concentrations of H_2_O_2_ at different time points and cell viability analysis through MTT assay on absorbance at 570 nm. ^∗^*p* < 0.05 vs. control group. (b) Cell viability evaluation through MTT assay following HLECs treated with KGF-2 at varying concentrations for 24 h. ^∗^*p* < 0.05 vs. control group. (c) Cell viability evaluation through MTT assay on HLECs pretreated using KGF-2 for 2 h, and 100 *μ*M H_2_O_2_ was added for treatment for 12 h. ^∗^*p* < 0.05 vs. control group. #*p* < 0.05 vs. cell treated with H_2_O_2_ alone. Data are presented in a form of mean ± SD from 3 separate assays.

H_2_O_2_ damaged cell viability in a concentration and time-dependent way (Figure 1(a)). 100 *μ*M H_2_O_2_ for 12 h was used in the following tests, which decreased cell viability to around 53.22 ± 1.29% in comparison with controls (without H_2_O_2_ treatment). Treatment with a broad range of KGF-2 concentrations exerted no cytotoxic effects on HLECs (Figure 1(b)). Besides, HLECs pretreated by KGF-2 exhibited a concentration-dependent protective effect against H_2_O_2_ damage (Figure 1(c)).

### 3.2. KGF-2 Suppressed H_2_O_2_-Induced Apoptosis in HLECs

**Figure 2:**
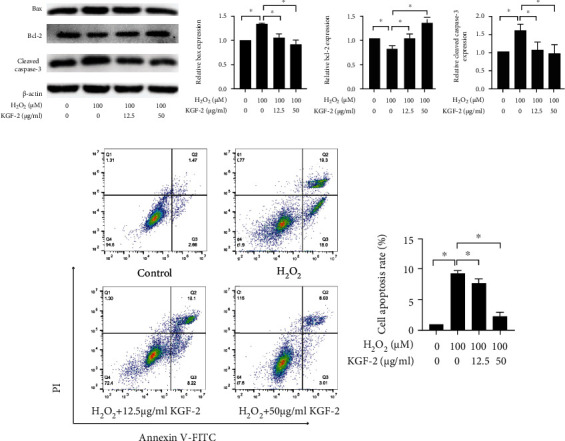
KGF-2 relieved H_2_O_2_-induced apoptosis in HLECs. (a) Protein level detection through western blotting analysis for Bax, Bcl-2, and cleaved caspase-3 of treated HLECs. (b) The proportion of apoptosis was measured by Annexin V-FITC and PI assays with flow cytometry. Data are presented in a form of mean ± SD from 3 separate assays. ^∗^*p* < 0.05.

Bcl-2 protein family has a critical function in modulating the mitochondrial apoptotic pathway [32]. In addition, the caspase family exerts a crucial function in the initiation and execution of programmed cell death. As a kind of executioner caspase, caspase-3 is activated within apoptotic cells via the internal and external (death ligand) pathways [33]. Therefore, members in the Bcl-2 family (either proapoptotic or antiapoptotic) and cleaved caspase-3 can affect the performance of apoptosis. Western blotting analysis revealed that exposure to H_2_O_2_ enhanced Bax and cleaved caspase-3 expression but reduced Bcl-2 expression in HLECs, whereas such effects could be eliminated through pretreatment with KGF-2 in a concentration-dependent manner (Figure 2(a)). Consistent with western blotting analysis, flow cytometry manifested that KGF-2 could relieve H_2_O_2_-induced viability decrease of HLECs (Figure 2(b)).

### 3.3. KGF-2 Inhibited H_2_O_2_-Induced Oxidative Stress in HLECs

**Figure 3:**
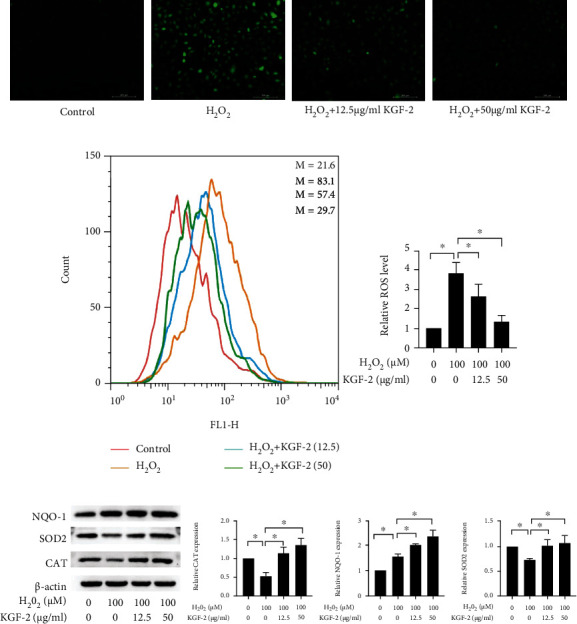
KGF-2 hindered H_2_O_2_-induced oxidative stress of HLECs. HLECs were treated with 12.5 and 50 *μ*g/mL KGF-2 for 2 h before incubation with 100 *μ*M H_2_O_2_ for 12 h. (a) ROS production was measured with 10 *μ*M DCFH-DA after treatment of the cells. The morphological characteristics in the cell were analyzed under fluorescence microscopy. (b) ROS production was examined by flow cytometry. (c) NQO-1, SOD2, and CAT protein levels could be observed through western blotting analysis. Results are presented in a form of mean ± SD from 3 separate assays. ^∗^*p* < 0.05.

HLECs exposed to H_2_O_2_ have a light green color after the incubation of the DCFH-DA reagent, indicating a prominent rise of ROS levels. Such rise could be avoided in a concentration-dependent way by pretreatment with KGF-2 (Figure 3(a)). Flow cytometry analyses reveal that H_2_O_2_-induction markedly elevated intracellular ROS production in comparison with control, while KGF-2 remarkably reduced it compared with H_2_O_2_-induced HLECs (Figure 3(b)). Furthermore, the antioxidant capacity of KGF-2 in H_2_O_2_-stimulated HLECs is assessed by measuring the levels of antioxidant enzymes (containing SOD2, NQO-1, and CAT). H_2_O_2_ significantly reduces SOD2 and CAT activity and increases NQO-1 content relative to the control group. In comparison with the H_2_O_2_ group, KGF-2 pretreatment substantially increases SOD2 and CAT levels and further increases NQO-1 content (Figure 3(c)). These findings show that KGF-2 inhibits H_2_O_2_-induced oxidative damage of HLECs.

### 3.4. KGF-2 Treatment Reduces Oxidative Stress and Apoptosis by Activating the PI3K/Akt Pathway

**Figure 4:**
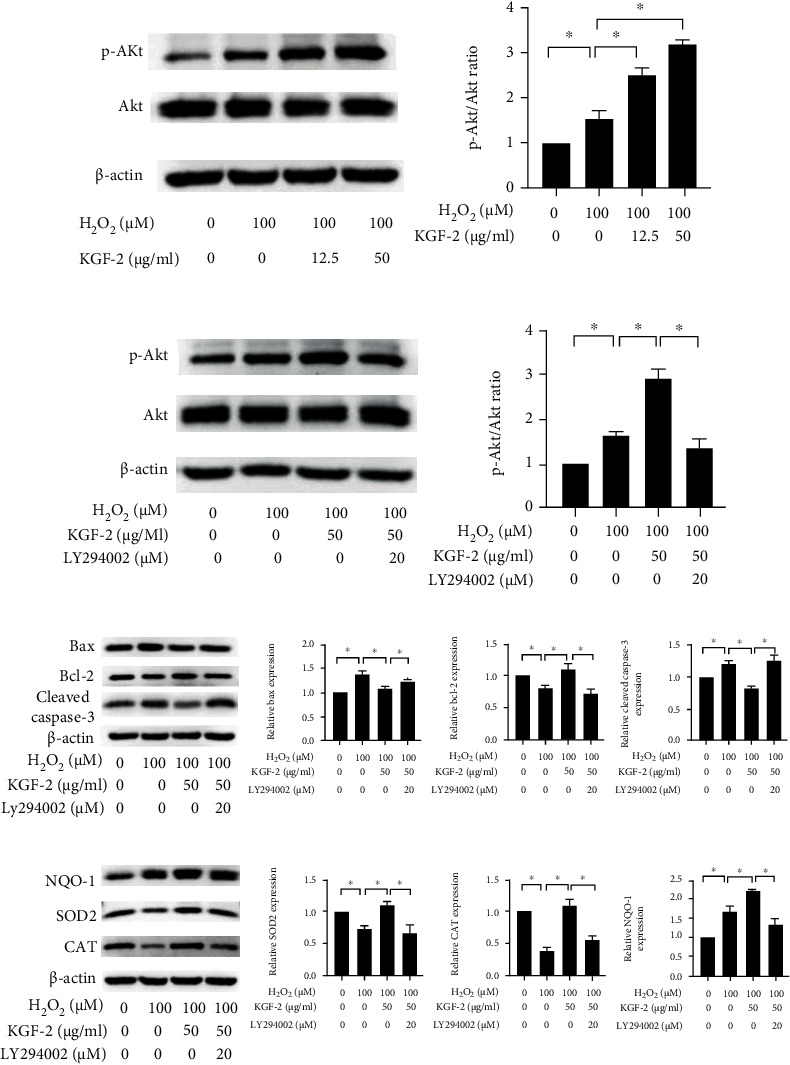
KGF-2 inhibited H_2_O_2_-induced apoptosis and oxidative stress by eliciting the PI3K/Akt pathway. HLECs were treated with or without KGF-2 for 2 h and were subsequently added with 100 *μ*M H_2_O_2_ for 12 h. (a) p-Akt and Akt protein levels of treated HLECs were measured through western blotting. Before adding KGF-2 and H_2_O_2_, HLECs were pretreated with 20 *μ*M LY294002 for 2 h. (b) Western blotting analysis quantified Akt and p-Akt levels. (c) Western blotting analysis quantified cleaved caspase-3, Bcl-2, and Bax levels. (d) Western blotting analysis quantified NQO-1, CAT, and SOD2 levels. Results are presented in a form of mean ± SD from 3 separate assays. ^∗^*p* < 0.05.

KGF-2 facilitates multiple biological activities via paracrine through activating PI3K/Akt pathway [21, 34]. Western blotting analysis indicated KGF-2 significantly reinforced phosphorylation in Akt in a concentration-dependent manner (Figure 4(a)). In contrast, LY294002, an inhibitor for PI3K/Akt, suppresses the Akt phosphorylation induced by KGF-2 (Figure 4(b)). We also used LY294002 to verify if the PI3K/Akt pathway contributed to KGF-2-mediated cytoprotective effects. LY294002 could reverse the antiapoptotic effect of KGF-2 through increasing Bax and cleaved caspase-3 content and decreasing Bcl-2 content compared with KGF-2 pretreatment (Figure 4(c)). Meanwhile, LY294002 attenuated KGF-2-induced increase of the activates of SOD2, CAT, and NQO-1 content in H_2_O_2_-stimulated HLECs (Figure 4(d)). These findings demonstrate that KGF-2 suppressed H_2_O_2_-induced apoptosis and oxidative stress of HLECs by eliciting the PI3K/Akt pathway.

### 3.5. KGF-2 Activates the Nrf2/HO-1 Signaling Pathway via the PI3K/Akt Pathway and Involves against H_2_O_2_-Induced Cytotoxicity

**Figure 5:**
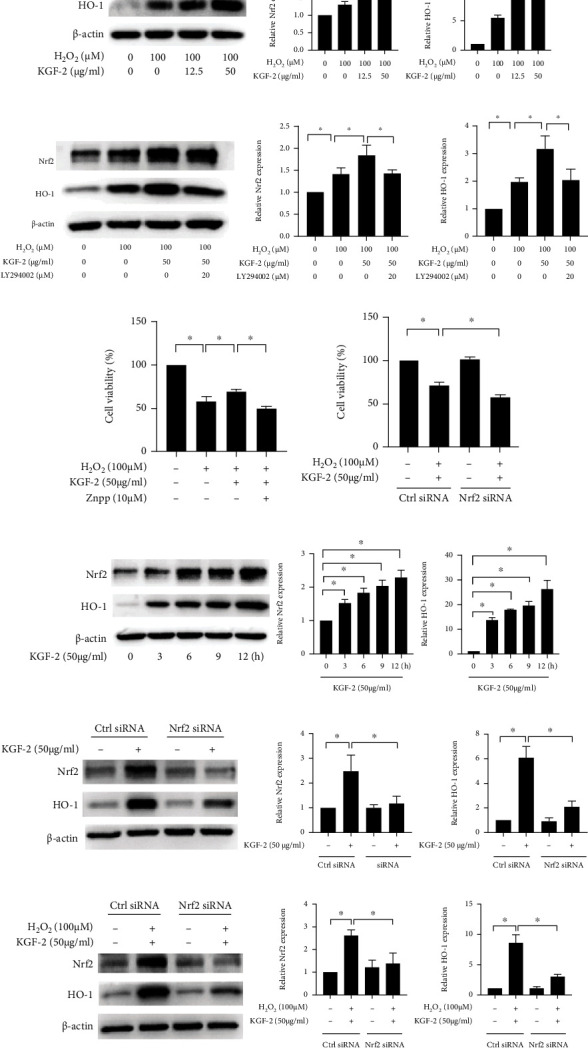
KGF-2 affects Nrf2 and HO-1 levels by the PI3K/Akt pathway and demonstrates cytoprotective effects via the Nrf2/HO-1 pathway. (a) HLECs were pretreated with or without KGF-2 for 2 h, followed by H_2_O_2_ treatment for 12 h. Western blotting analysis quantified Nrf2 and HO-1 levels. (b–d) HLECs were pretreated with 20 *μ*M LY294002 for 2 h or 10 *μ*M ZnPP for 30 min or Nrf2 siRNA for 12 h, followed by 2 h treatment with KGF-2 and further 12 h treatment with H_2_O_2_. Western blotting analysis quantified Nrf2 and HO-1 levels, and MTT assay evaluated cell viability. (e) HLECs were pretreated with KGF-2 alone at different times. (f) HLECs were subject to 12 h transient transfection by Nrf2 siRNA and then 12 h KGF-2 treatment. (g) HLECs were subjected to 12 h transient transfection using Nrf2 siRNA, then 2 h KGF-2 treatment, and 12 h H_2_O_2_ treatment. Western blotting analysis quantified Nrf2 and HO-1 levels. Results are presented in a form of mean ± SD from 3 separate assays. ^∗^*p* < 0.05.

This study analyzed the effects of KGF-2 on the Nrf2/HO-1 pathway to explore the molecular mechanism further. The western blotting analysis demonstrates that KGF-2 pretreatment efficiently promotes Nrf2 and HO-1 protein expression in comparison with the H_2_O_2_-stimulated HLECs (Figure 5(a)). Moreover, LY294002 can block Nrf2 and HO-1 protein expression induced by KGF-2 (Figure 5(b)), indicating that LY294002 significantly inhibits the cytoprotective effect of KGF-2.

To verify whether HO-1 and Nrf2 were required for the protective function of KGF-2, we pretreated HLECs with ZnPP and evaluated cell viability by MTT assays. ZnPP remarkably reversed the cytoprotective impacts of KGF-2 on H_2_O_2_-induced cytotoxicity (Figure 5(c)). Besides, Nrf2 siRNA has significantly weakened the cell viability in the HLECs pretreated by KGF-2 (Figure 5(d)). According to the obtained data, Nrf2 and HO-1 exert a crucial function in the cytoprotection regulated by KGF-2. Then, we studied the impacts of KGF-2 alone on Nrf2 and HO-1 induction to verify if it is directly linked to both activations. HLECs treated with KGF-2 alone led to time-dependent induction in Nrf2 and HO-1 protein expression (Figure 5(e)). The Nrf2 siRNA treatment notably abolished the levels of HO-1 expression regulated by KGF-2 in KGF-2-induced HLECs, suggesting that the upregulation of HO-1 is elicited by KGF-2 mediation *via* Nrf2 activation (Figure 5(f)). Furthermore, we investigated the relationship between the Nrf2 and the HO-1 handled by KGF-2 in H_2_O_2_-stimulated HLECs. Similarly, preincubation with Nrf2 siRNA markedly suppressed KGF-2-mediated upregulation of Nrf2 and HO-1 at the protein levels (Figure 5(g)). These findings support the fact that the cytoprotective effects of KGF-2 were mediated by the Nrf2/HO-1 pathway.

### 3.6. KGF-2 Prevents H_2_O_2_-Induced Lens Opacity

**Figure 6:**
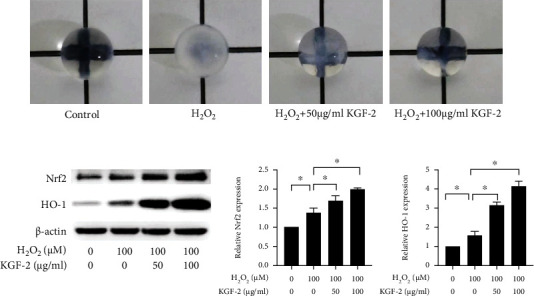
Effect of KGF-2 on lens organ incubate. (a) Effects of KGF-2 on the transparency of H_2_O_2_-induced rat lens. (b) Measurement of protein levels in lenses of Nrf2 and HO-1 using the western blotting analyses. Results are presented in a form of mean ± SD from 3 separate assays. ^∗^*p* < 0.05.

An organ culture experiment was performed to examine how KGF-2 affected cataractogenesis of rat lenses induced with H_2_O_2_. Morphological observational results verified transparency loss of lenses under exposure to H_2_O_2_. By contrast, untreated lenses showed almost no change. The opacities of rat lenses incubated in H_2_O_2_ media containing various concentrations of KGF-2 were analyzed and show remission in a KGF-2 concentration-dependent manner (Figure 6(a)). Western blotting measures Nrf2 and HO-1 level in H_2_O_2_-stimulated lens. Pretreatment with KGF-2 significantly increases the expression of Nrf2 and HO-1 (Figure 6(b)). Accordingly, KGF-2 blocked the process of cataractogenesis.

## 4. Discussion

Cataracts are the foremost cause of blindness globally, bringing serious socioeconomic crises to numerous countries. Unfortunately, there still lacks a well-received pharmacological agent to suppress opacification. Oxidative stress, characterized by excessive ROS, is an essential mediator of both the initiation and the progression of cataracts. Excessive production of ROS may disrupt oxidation-antioxidant balance and cause mitochondrial dysfunction, lipid peroxidation, or cell apoptosis. Thus, decreasing oxidative stress and avoiding oxidative stress-induced apoptosis are likely therapeutic targets for cataracts [35].

KGF-2, a typical paracrine growth factor, signals using the interaction with its high-affinity receptor FGFR2-IIIb splicing isoform [36]. KGF-2 has numerous biological functions, including regulating cell survival and proliferation, suppressing inflammation, or exhibiting antioxidant effects. The exogenous supplementation of KGF-2 prevents the formation and development of multiple illnesses, such as wound healing defects, cardiovascular diseases, and metabolism syndrome, as well as acute kidney injury [18, 37–39]. For example, KGF-2 encourages axonal regeneration and functional recovery following peripheral nerve injury through protecting Schwann cells against excessive oxidative stress-induced apoptosis [23]. Our study reveals that KGF-2 exhibits no apparent cytotoxicity in HLECs. Given its nontoxic properties and favorable antioxidative effects, using KGF-2 to treat age-related cataracts may be an effective way.

H_2_O_2_, as an essential component in ROS, can accumulate considerably in both lens and aqueous humor [6]. Evidence had demonstrated the role of H_2_O_2_ in triggering HLECs apoptosis, causing the initiation of cataract formation [40]. The research showed exposure to concentration-dependent H_2_O_2_ decreased cell viability of HLECs, indicating H_2_O_2_ cytotoxicity against HLECs. However, pretreatment with KGF-2 relieved H_2_O_2_-induced decrease of HLEC viability and meantime prohibited apoptosis of HLECs induced by H_2_O_2_. Bcl-2, Bax, and cleaved caspase-3's proteins have been considered the foremost regulators of apoptosis. Bax is a proapoptotic gene, while Bcl-2 belongs to an antiapoptotic gene [41]. Caspase-3 is the most used target to detect apoptosis [42]. H_2_O_2_ leads to Bcl-2 downregulation whereas Bax and cleaved caspase-3 upregulation, but the downregulation of Bcl-2 and the upregulation of Bax and cleaved caspase-3 were reversed after treatment with different concentrations of KGF-2. Besides, data indicate that KGF-2 has no cytotoxicity on HLECs. In general, KGF-2 exerts cytoprotective effects through ameliorating cell viability and blocking apoptosis of oxidative stress-stimulated HLECs.

Intracellular ROS is an indicator that reflects the level of oxidative stress directly. Hence, effects of KGF-2 on H_2_O_2_-induced intracellular ROS using the flow cytometry technique were detected. The results revealed that KGF-2 inhibits intracellular ROS production, showing the obvious antioxidative effects of KGF-2. In the meantime, our studies also showed an enhancement in the endogenous antioxidant systems with KGF-2 treatment. The antioxidant systems include numerous antioxidant enzymes including SOD2, CAT, and NQO-1, protecting cells from oxidative impairment following oxidative insult or during physiological metabolism. The three enzymes are indispensable to normal lens metabolism, which protect the lens against oxidative stress and retain lens transparency [43, 44]. H_2_O_2_-induced cataracts were linked to reduced SOD2 and CAT activities and reinforced NQO-1 activity of the lens. These changes were facilitated by KGF-2 at varying concentrations, proving that KGF-2 has the function of alleviating oxidative stress through increasing endogenous antioxidants.

The PI3K/Akt pathway provides a critical pathway cascade for promoting cell survival and inhibiting apoptosis. Furthermore, research shows that activation via the PI3K/Akt pathway promotes the antioxidant defense ability of HLECs [45]. Considering that PI3K/Akt pathway has an essential effect on preventing cell stress and enhancing cell survival, it is speculated that this pathway is probably the critical pathway for KGF-2 to exert its cytoprotective effect. In our study, KGF-2 remarkably promoted phosphorylation in Akt subject to oxidative stress conditions, indicating that KGF-2 elicited the PI3K/Akt pathway to resist oxidative stress and retain cell survival. For deepening existing research on PI3K/Akt pathway and its protective effects on KGF-2 against H_2_O_2_-induced HLEC injury, LY294002, a pharmacological inhibitor of PI3K/Akt pathway engaged with the cytoprotective effects of KGF-2, was studied. LY294002 reversed the change of the antioxidant enzymes (SOD2, CAT, and NQO-1) and the apoptotic regulators (Bcl-2, Bax, and cleaved caspase-3) induced by KGF-2 in the H_2_O_2_-stimulated HLECs. It also significantly decreased SOD2, CAT, and Bcl-2 expression and increased NQO-1, Bax, and cleaved caspase-3 expression, demonstrating PI3K/Akt pathway's involvement in cytoprotective effects on KGF-2.

It is universally believed that Nrf2 is a significant transcription factor that plays an indispensable role in the expression of antioxidant and detoxification enzymes. HO-1, a famous phase II detoxifying enzyme, can considerably balance redox homeostasis and inhibit inflammatory illnesses [46, 47]. In oxidative stress, HO-1 undergoes upregulation to reinforce the cellular defense mechanism against oxidative insult [48–50]. PI3K/Akt pathway is found to exert a cytoprotective influence by enhancing the Nrf2/HO-1 pathway [51–54]. Data shows that KGF-2 pretreatment effectively increases the expression of Nrf2 and HO-1 in H_2_O_2_-stimulated HLECs. However, this increase was significantly downregulated by LY294002, indicating the partial involvement of the PI3K/Akt pathway in activating the Nrf2 and HO-1. An interesting finding is that ZnPP and Nrf2 siRNA abolishes the KGF-2's protective effect against H_2_O_2_-induced HLEC death, indicating that Nrf2 and HO-1 are essential in KGF-2-induced cytoprotection. Besides, Nrf2 belongs to an upstream mediator of phase II detoxifying enzymes; thus, Nrf2 translocation can mediate HO-1 gene transcription for enhancing cellular defensive capacity and combating oxidative stress [55, 56]. We evaluated the effect of KGF-2 alone on Nrf2 and HO-1 to further demonstrate that KGF-2 participates in the activation of a cytoprotective pathway. The results demonstrated that KGF-2 activates HO-1 and Nrf2 levels depending on time. When we knock down the expression of Nrf2 using Nrf2 siRNA, the content of HO-1 is also reduced. We further determine the impact of KGF-2 on the expression of Nrf2 and HO-1 protein in H_2_O_2_-induced HLECs. Similarly, Nrf2 siRNA abolished this enhancement in Nrf2 and HO-1 induced by KGF-2 in H_2_O_2_-stimulated HLECs, suggesting the cytoprotective effect of KGF-2 in regard to the Nrf2/HO-1 pathway. Therefore, these findings provide direct evidence that the Nrf2/HO-1 pathway regulated by KGF-2 may be an underlying therapeutic target for cataracts.

The *in vitro* experiments confirmed the effects of KGF-2 on the prevention of oxidative stress-induced cell apoptosis, whereas the key to the problem is whether KGF-2 is efficient in lens tissue. For answering the question, we refer to the findings in an *ex vivo* experiment evaluating KGF-2's anticataract potential of H_2_O_2_-mediated rat lens isolation through monitoring the lens transparency while predicting several biochemical variables like Nrf2 and HO-1 contents. Compared with the normal lenses, the transparency of the lens exposed to H_2_O_2_*in vitro* was decreased. However, the H_2_O_2_-induced opacity of lenses was ameliorated by treatment using KGF-2. Therefore, results in relevant ex vivo experiments suggest that KGF-2 effectively stunted H_2_O_2_-induced cataract formation, possibly by increasing the contents of Nrf2 and HO-1 to maintain the lens transparency.

In conclusion, this study confirmed the ability of H_2_O_2_ in inducing HLEC apoptosis and lens opacification, while KGF-2 may efficiently alleviate lens opacity and the apoptosis of human lens epithelial cells under H_2_O_2_-induced oxidative stress. Furthermore, KGF-2, an effective antioxidant, was found to be efficiently protect HLECs through PI3K/Akt and Nrf2/HO-1 signaling pathways (see supplementary materials (available here)). Also, the safety of KGF-2 in intraocular use has been confirmed; we believed that KGF-2 can be applied in clinical practice as an underlying protective agent in cataract formation.

## Data Availability

All data included in this study are available upon request by contact with the corresponding author.

## Supplementary Materials

Supplementary Materials A schematic diagram of the pathway by which KGF-2 protected HLECs from H_2_O_2_-induced oxidative stress.
