# Supplementary figures and images for: Protection of Human Lens Epithelial Cells from Oxidative Stress Damage and Cell Apoptosis by KGF-2 through the Akt/Nrf2/HO-1 Pathway
Source: Oxid Med Cell Longev. 2022 Feb 17;2022:6933812. doi: 10.1155/2022/6933812 (PMC8872674; doi:10.1155/2022/6933812)

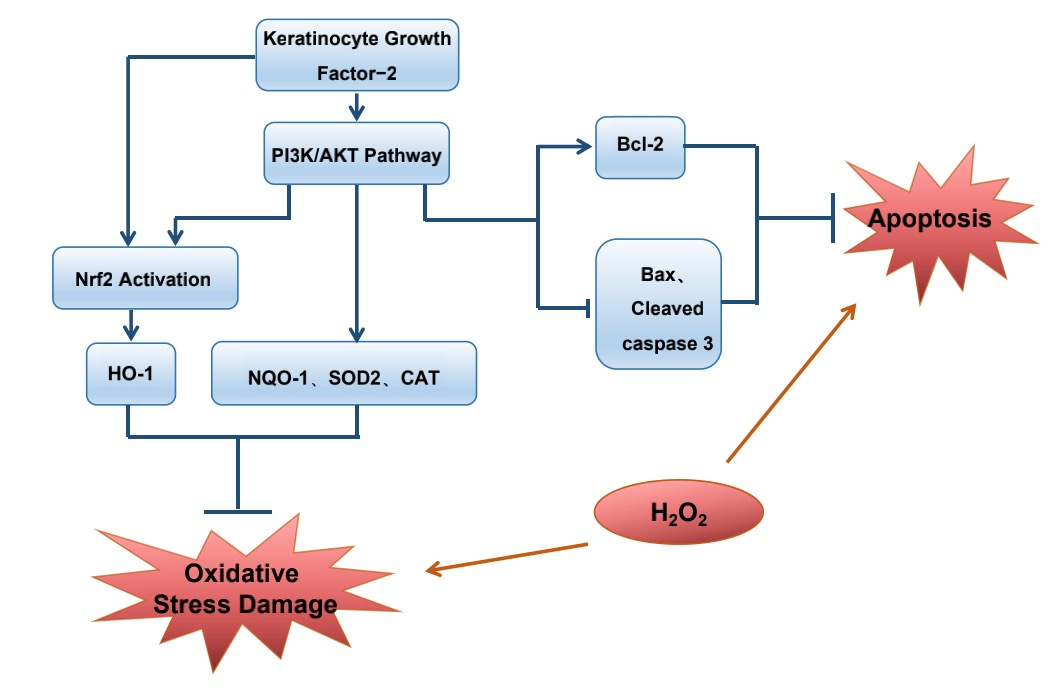

Supplement: Supplementary Materials — A schematic diagram of the pathway by which KGF-2 protected HLECs from H2O2-induced oxidative stress. [file 6933812.f1.png]
